# Seed-mediated synthesis and SERS performance of graphene oxide-wrapped Ag nanomushroom

**DOI:** 10.1038/s41598-017-10262-9

**Published:** 2017-08-29

**Authors:** Tao Jiang, Xiaolong Wang, Shiwei Tang, Jun Zhou, Chenjie Gu, Jing Tang

**Affiliations:** 1 Institute of Photonics, Department of Microelectronic Science and Engineering, Faculty of Science, Ningbo University, Ningbo, 315211 P. R. China; 2 Institute of Physics, Ningbo University of Technology, Ningbo, 315016 P. R. China

## Abstract

A facile seed-mediated method was developed to modify core-shell Ag nanosphere@PSPAA with another Ag layer for achieving an enhancement of their surface-enhanced Raman scattering (SERS) activity. Interestingly, an Ag bridge in the polymer shell connected the inner and outer Ag layers, resulting in a mushroom-like nanostructure. The outer Ag grew around the polymer shell to form the cap of the nanomushrooms (NMs) with the extension of the reaction time. The epitaxial growth mechanism of this novel nanostructure was investigated by tuning the type of seed from nanosphere to nanocube and nanorod. With the growth of the outer Ag cap, the SERS intensity of these Ag NMs increased significantly together with the red-shifting and broadening of their typical localized surface plasmon resonance band. Such a phenomenon can be attributed to the formation of SERS hotspots between the inner and outer Ag layers. The Ag NMs were then wrapped with a graphene oxide (GO) shell via static interactions. The GO-wrapped Ag NMs exhibited a further better SERS performance in terms of sensitivity, homogeneity and stability compared with non-wrapped ones, indicating that the heterostructure could be potentially useful for SERS-based immunoassay.

## Introduction

Over the past two decades, surface-enhanced Raman scattering (SERS) has evolved into a notably promising analytical method in the fields of life care, environmental monitoring, food safety, and state security due to its single-molecule sensitivity^1–5^. Among the various SERS-based materials, noble metal nanostructures have demonstrated superior activity since the generation of extremely strong electromagnetic field under the excitation of their localized surface plasmon resonance (LSPR)^6–8^. Among all these noble metals, Ag stands out due to its easy fabrication process and excellent SERS performance^9^. Recent advances in wet-chemical synthesis further enable the fabrication of various Ag nanostructures with engineered complex size, shape, and composition, such as nanorods^10^, nanoplates^11^, nanocubes^12^, and nanostars^13^. These nanostructures have been extensively prepared by using different types of surfactant to effectively tune their plasmonic properties. Nevertheless, these Ag nanostructures suffer from the tendency to oxidation, possessing serious limitations for their use as reliable long-term SERS substrates^14^. Particularly, the efficiency and reliability of the SERS nanoprobes are often compromised by ligand dissociation or exchange^15^. In addition, the SERS signal could be easily influenced by variations of practical environments. A variety of coating materials were, therefore, introduced to protect the exposed Ag surface and enhance the stability of the nanoprobes, such as carbon^16^, Au^17^, silica^18^, and polymer^15^. Among all of them, amphiphilic diblock copolymer as an encapsulation material has been widely studied. For example, polystyrene-block-poly(acrylic acid) (PSPAA), which can form a shell with diameter of about 10 nm around the nanoparticles (NPs), provide a good protection of the Ag^19^. The main advantage of such an ultra-thin polymer shell is good light transmission without affecting the output of SERS signals. On the other hand, SERS signals of individual NPs without gaps in assemblies are usually too weak to be used in biochemical assay, although the signals can also generate in presence of local electromagnetic fields^20–22^. Therefore, some novel noble metal structures full of intermetallic gaps and sharp edges such as nanomushrooms have attracted great attentions in SERS^23, 24^. If the ultra-thin polymer shell could be used to as a spacer between two noble metal layers, the plasmonic coupling between them will then induce intense electromagnetic hotspots. Compared with the single NP without gap, extreme enhancement of SERS signal from the molecules in the polymer shell can be expected^25, 26^. Fortunately, the exposed hydrophilic functional group on the surface of PSPAA shell can easily adsorb Ag ions to form an outside Ag shell under the effect of reducer, which might be effectively utilized to construct SERS hotspots.

What’s more, other than the high intensity of output signal, other performances such as reproducibility, uniformity, and stability are also crucial to the practical application of SERS substrates. However, the lack of criterions for these characters have resulted in few successful attempt to fabricate suitable SERS substrates^27, 28^. Thus, the design and preparation of low-cost SERS substrates with good homogeneity and long-term stability are still highly desired. Consequently, various hybrid structures of noble metal and other materials including semiconductor and graphene have been exploited to serve as new kinds of SERS substrate^29, 30^. Graphene, as a two-dimensional nanomaterial, is chemically inert, highly impermeable to ambient oxygen, transparent to a broad span of light and can even magnify the SERS signal, which make the composite of noble metal and graphene an excellent candidate for SERS substrate^31, 32^. As one of the most important graphene derivatives, graphene oxide (GO) has superior bio-compatibility and chemical stability due to a large quantity of hydrophilic oxygenated functional groups. These groups are much more significant for selective adsorption of molecules and output of stable SERS signal with relatively better homogeneity^33^. To date, the most common method for the preparation of GO supported or covered noble metal substrates focus on simply transferring pre-synthesized GO under or onto a layer of noble metal NPs^34–39^. Only a few groups demonstrated GO-wrapped Au or Ag NPs for SERS applications due to the synthesis barrier^40^. With the fast development of nanotechnology, the design and fabrication of novel core-shell nanostructures to well control their physical properties have attracted considerable attention. As a consequence, it is highly desirable to develop other types of GO-wrapped Ag nanostructure for obtaining much better SERS performance.

In this study, a seed-mediated growth method was applied to synthesize Ag nanomushrooms (NMs). Monodispersed Ag nanospheres (NSs) of about 20 nm in diameter were first synthesized and encapsulated by PSPAA using SERS-active ligand (2-naphthalenethiol, 2NT) to form core-shell Ag NS@PSPAA nanostructures as a core. Then, Ag NPs with a mushroom-like shape was prepared through the growth of an outside Ag layer on the core by *in situ* reducing under the assistance of surfactant sodium citrate. A temporal dependent experiment was performed to monitor the growth process of the Ag NMs and NMs with caps of different size were obtained. Besides, the growth mechanism of this novel nanostructure was further studied by changing the Ag seed from NS to nanocube and nanorod, followed by the control experiments using bare Ag NSs as seed or without seed. Compared with the initial Ag NS@PSPAA, intenser SERS signals were observed from these Ag NMs due to the formation of hotspots within the much narrow polymer gap. The SERS intensity of the Ag NMs increased gradually with the growth of the Ag cap and the enhancement ratio of SERS signal from the final sample (60 min) to that from the Ag NS@PSPAA was 3.89. Thereafter, we employed a static interaction method to encapsulate the Ag NMs into a multilayer GO shell. The experimental results show that SERS signals from the GO-wrapped Ag NMs were about 2 times higher than those from the bare NMs. In addition, the wrapping of GO simultaneously improved the homogeneity of the SERS probe, showing an intensity mapping with RSD of only 13.22%. The excellent long-term stability of the GO-wrapped Ag NMs was finally compared with the unwrapped ones. After exposing to ambient air for one month, the GO-wrapped sample exhibited a more stable SERS signal with decrease ratio of only 15%.

## Methods

### Materials

Hydrogen tetrachloroaurate(III) hydrate (HAuCl_4_∙xH_2_O, 99.9%), sodium citrate, AgNO_3_, sodium borohydride (NaBH_4_), L-ascorbic acid, 2NT, and cysteamine hydrochloride were purchased from Sigma-Aldrich. Amphiphilic diblock copolymer PSPAA (PS_154_-b-PAA_49_, M_n_ = 16000 for the PS block and M_n_ = 3500 for the PAA block, M_w_/M_n_ = 1.15) was obtained from Polymer Source. Milli-Q water (resistance > 18.2 MΩ·cm^−1^) was used throughout the whole experiment.

### Synthesis of Ag NSs

The synthesis begins with the preparation of citrate-stabilized seed NPs (see Supplementary material). The seed solutions were used within 2–5 hrs after the preparation. A 50 mL solution of AgNO_3_ (10 mg) and sodium citrate (30 mg) were then prepared. The seed solution (3 mL) was added quickly with vigorous stirring. Then, a solution of ascorbic acid (20 mg in 10 mL water) was added dropwise for about 5 min, and the mixture was stirred for an additional 1 h. A brownish-yellow solution of Ag NSs was obtained.

### Encapsulation of Ag NSs with PSPAA

The single encapsulation of Au or Ag NPs by PSPAA using SERS-active ligands has been previously reported^15^. The detailed synthesis process can be found in Supplementary material.

### Synthesis of Ag NMs

The above Ag NS@PSPAA was added to 10 mL aqueous solution of 6 mg sodium citrate and 2 mg AgNO_3_ with vigorous stirring. Subsequently, a solution of ascorbic acid (4 mg in 2 mL water) was added dropwise for about 1 h, and the mixture was stirred for an additional 1 h. The product was finally collected by centrifugation at 8000 rpm for 10 min.

### Synthesis of GO-wrapped Ag NMs

The GO solution (1 mg/mL) was firstly ultra-sonicated for 3 hrs. Next, 1 mL of as prepared Ag NMs were mixed with cysteamine and GO solutions to achieve final concentrations of 12 μM (cysteamine) and 0.01 mg/mL (GO). The mixture was left for 3 hrs under mild stirring at room temperature^40^.

**Figure 1:**
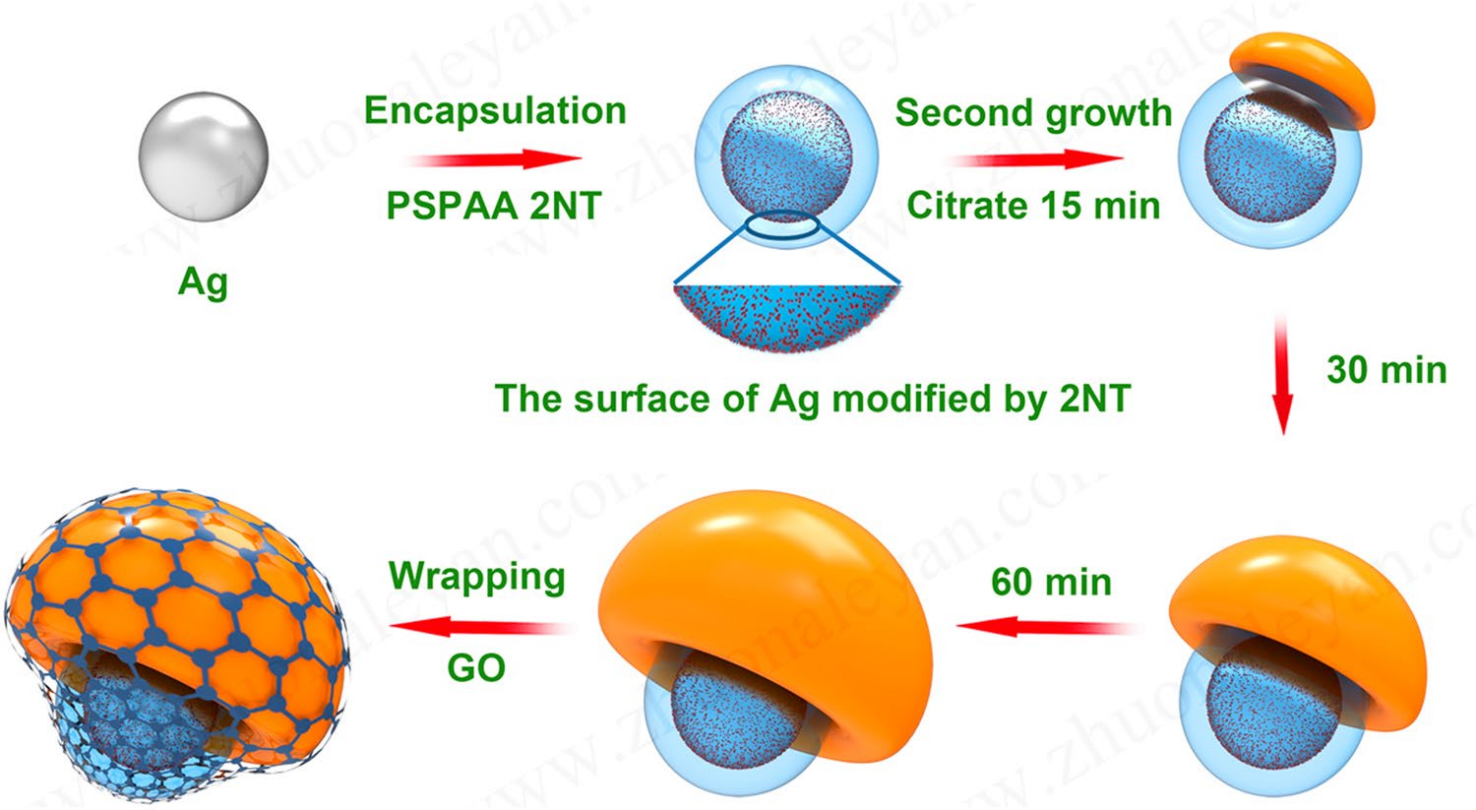
Schematic illustration of the seed-mediated approach for the graphene oxide-wrapped Ag NMs.

The synthesis strategy of the GO-wrapped Ag NMs is illustrated in Fig. 1.

### Characterization

The sizes and morphologies of the products were investigated by a SU-70 field emission scanning electron microscopy (FESEM) and a JEM-1400 transmission electron microscopy (TEM). The optical absorption spectra of the products were recorded with a Cary 100 UV-vis spectrophotometer. The SERS properties were recorded by a Raman spectrometer (Ntegra Spectra, NT-MDT) with the specifications as follows: a semiconductor laser of 532 nm wavelength and maximum power 20 mW, a 100× objective lens with numerical aperture (NA) of 0.7 to position the scanning probes and focus the laser beam, a 3D scanner with a resolution of 0.6 × 0.6 × 0.04 nm to response the morphology signal, an Al coated grating of 1800 lines/mm, and a charge-coupled device (CCD) (2048 × 2048 pixels) detector. The accumulation time was 10 s. All the analysis was performed at room temperature.

## Results

**Figure 2:**
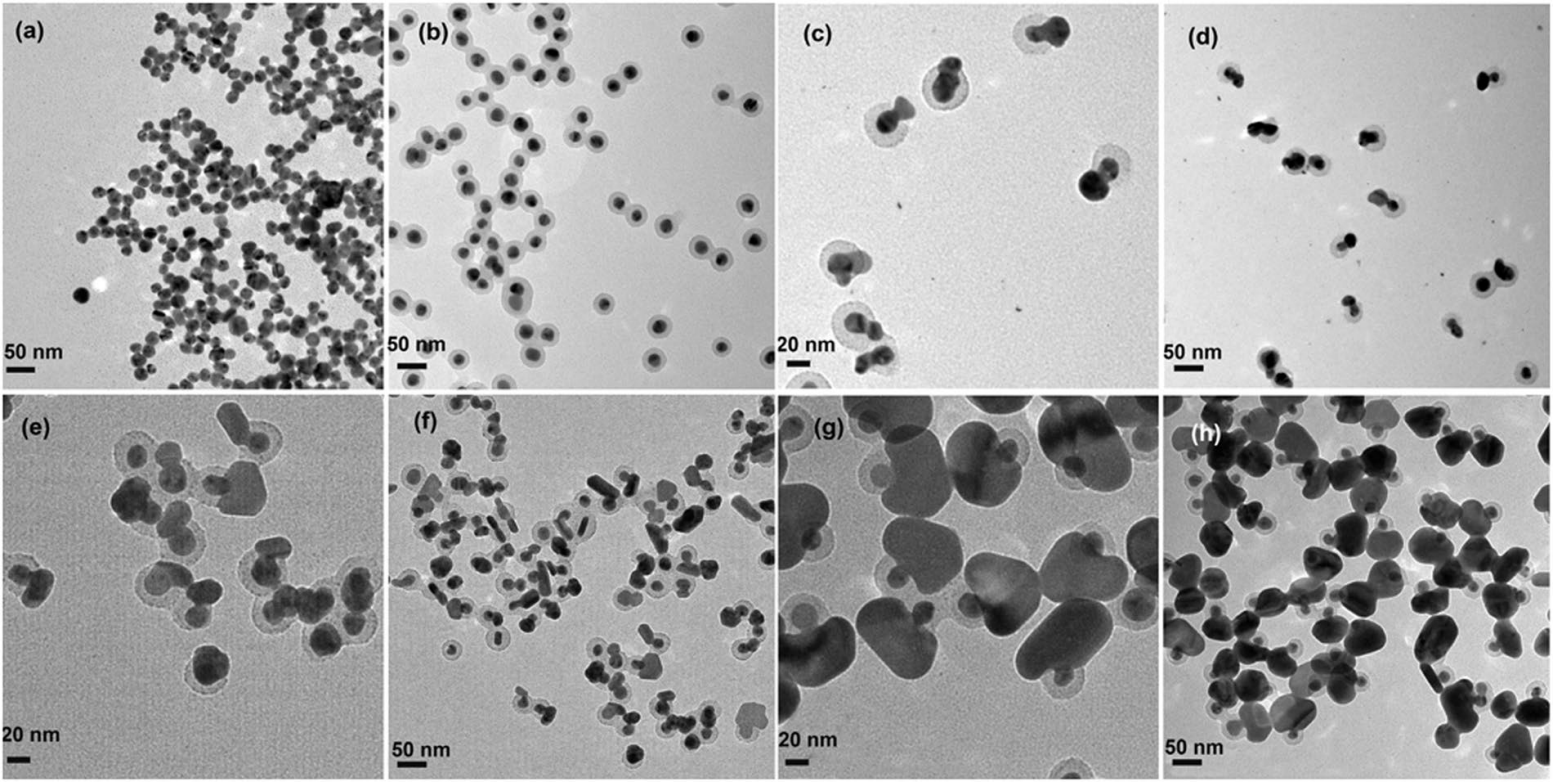
TEM images of (**a**) Ag NSs, (**b**) Ag NS@PSPAA, (**c**,**d**) Ag NMs (15 min), (**e**,**f**) Ag NMs (30 min), and (**g**,**h**) Ag NMs (60 min).

In this work, we used a modified seed-mediated growth method to synthesize Ag NMs. Monodisperse suspensions of Ag NSs with diameter of 20 ± 5 nm were first prepared as shown in Fig. 2a. After being encapsulated by PSPAA, the Ag NSs show ultrathin polymer shells with thickness of 10 ± 3 nm as presented in Fig. 2b. It should be pointed out that the Ag NSs was first decorated by ligand (2NT) and then encapsulated by the polymer shell in the heating process. Therefore, the location of the SERS-active 2NT molecules is on the surface of the Ag NSs and the distance between 2NT and the outside surface of the polymer shell is about 10 nm. Then, these core-shell NPs were used as seed for *in-situ* reducing AgNO_3_ on their surface to grow outside Ag layer. The growth process of the Ag layer was monitored as shown in Fig. 2c to h. Interestingly, it appears that the second grown Ag nucleates on the surface of the inner Ag and punches through the polymer shell. After that, the Ag domain gradually expands across the outside surface of the polymer shell, forming an uncompleted shell like a cap with the extension of reaction time. The shape of the Ag NP eventually merges into a mushroom-like structure with irregular cap. The size of the cap varies from 15 ± 5 nm, 30 ± 10 nm and finally to 60–100 nm throughout the growth process (15, 30, and 60 mins). In the polymer shell of some NMs, growth spots like a bridge connecting the inner and outer Ag can be clearly identified in Fig. 2c,e and g. However, in the final sample, the growth spots are not so obvious due to the fact that the Ag cap are big enough to completely cover the polymer shell on one side of the Ag NPs. The formation of interconnecting bridges due to the growth of Ag through the polymer shell suggests that the polymer block do not completely insulate the nucleation of Ag on the surface of the core NPs. According to previously reported rules for the epitaxial layered growth of core-shell NPs: the lattice constants of two metals should be comparable with the lattice mismatch smaller than about 5%^41^. Factually, there is no lattice mismatch between the inner and outer Ag, even the polymer shell rise a barrier, the epitaxial grow of the Ag cap can still happen effectively. In all the nanostructures, the existence of only one connecting spot can be attributed to that, if the second grown Ag nucleated at one position of the core surface, this position would hold the smallest growth energy barrier and the following Ag would not nucleate and grow on the other sites. The UV-vis spectra represent clearly the process of the encapsulation and growth of Ag NMs (Fig. S1). The absorption peak of the Ag NSs shifts from 402 to 436 nm after coated with the block copolymers because of the higher refractive index of the polymer, compared with water. With the growth of the Ag cap, the absorption band of samples become broaden significantly and the main peak further red-shifts from 436 to 475 nm gradually.

**Figure 3:**
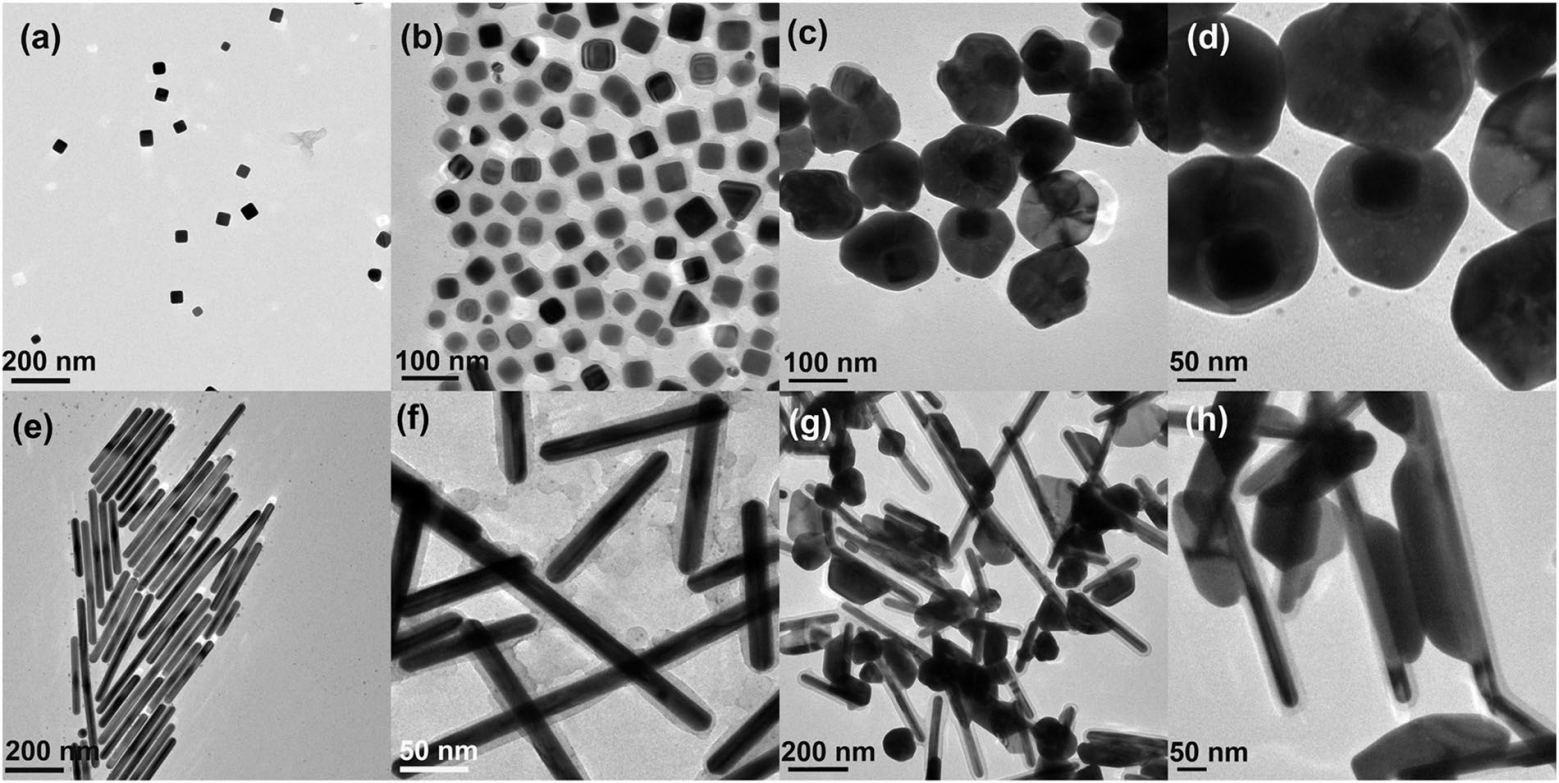
TEM images of (**a**) Ag nanocubes, (**b**) Ag nanocube@PSPAA, (**c**,**d**) Ag nanocube@PSPAA mediated Ag NMs, (**e)** Ag nanorods, (**f**) Ag nanorod@PSPAA, (**g**,**h**) Ag nanorod@PSPAA mediated Ag NMs.

It is noticed that the Ag cap maintains the plate shape, which is the typical character of the epitaxial growth mode. To certify this mechanism, we further applied Ag nanocube and nanorod encapsulated by polymer as the seed to grow Ag outside layer, respectively (Fig. 3). Despite the shape of the seed, the second grown Ag goes through the polymer shell to form heterostructures. The plate shape of the Ag shell is more obvious in the SEM image (Fig. S2) than that in the TEM image, which is particular true for the nanostructures with nanorod as seed. This feature confirms the epitaxial growth of the outside Ag layer. Consequently, we reason that epitaxial growth of Ag on the core at defected regions of the polymer lead to the formation of the nanobridges.

**Figure 4:**
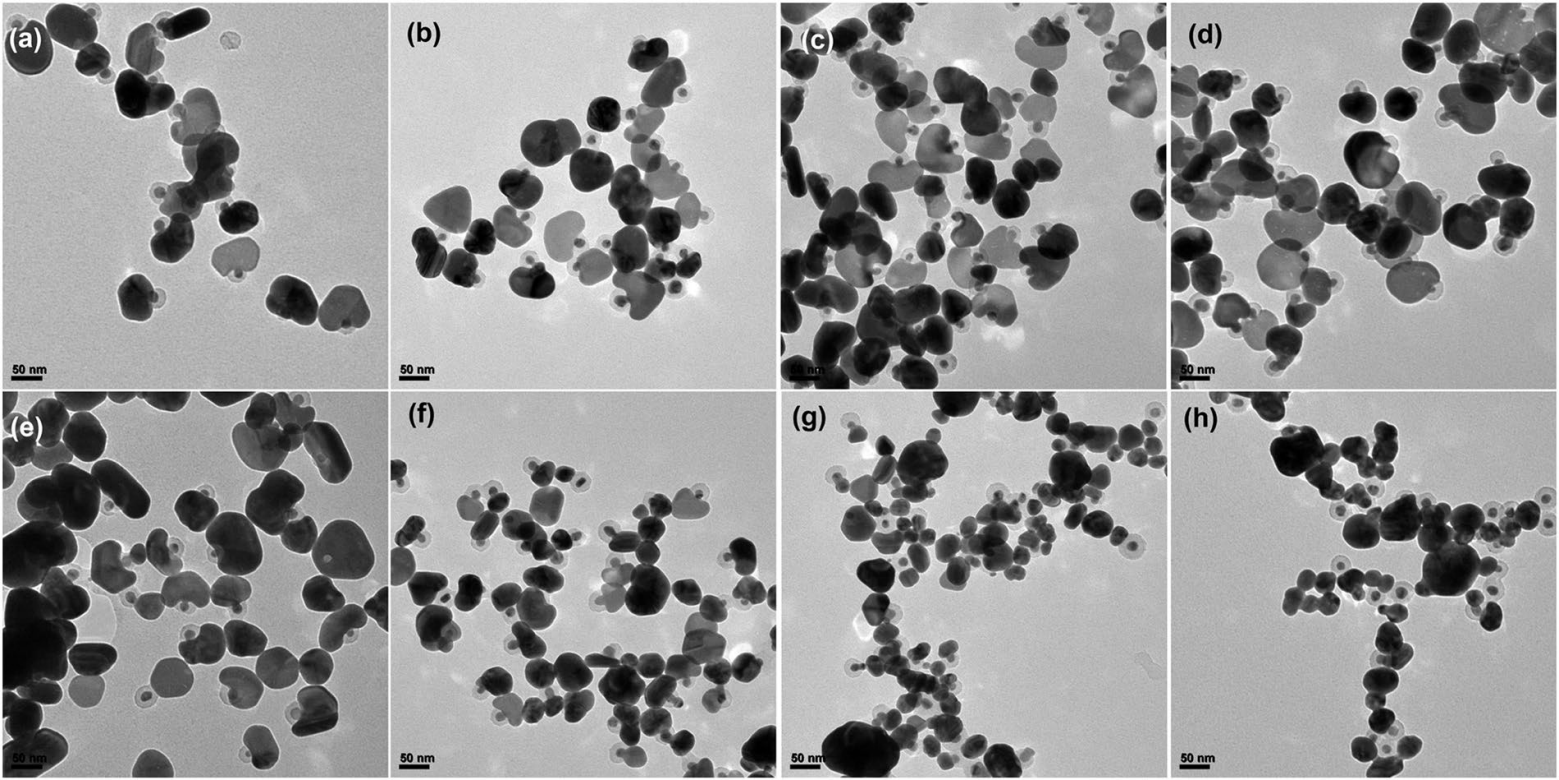
TEM images of Ag NMs synthesized by adding (**a**) 30, (**b**) 50, (**c**) 100, and (**d**) 150 μL of HNO_3_ (0.2 M) and (**e**) 30, (**f**) 50, (**g**) 100, and (**h**) 150 μL of NaOH (0.2 M) into the precursor solution, respectively.

The growth speed of the Ag cap was fully adjusted by adding various amount of acid or alkaline into the precursor solution. The results are presented in Fig. 4, which exhibits no substantial change of the plate shape of the as-prepared samples with the increasing of the acid (Fig. 4a to d). Nevertheless, the Ag caps with a quasi-spherical shape synthesized by adding alkaline are all smaller than those prepared using the same amount of acid (Fig. 4e to h). This is because that the reduction and growth of Ag is much slower in the acid environment than that in the alkaline solution. Therefore, the shapes of the final NPs are controlled more effectively by sodium citrate in the acid environment. According to the literature, sodium citrate usually adsorb on the (111) facet of Ag and result in a plate shape such as triangle nanoplates^42^. However, the second grown Ag did not form a completed shell probably due to the poor wetting effect of the Ag to the polymer surface.

**Figure 5:**
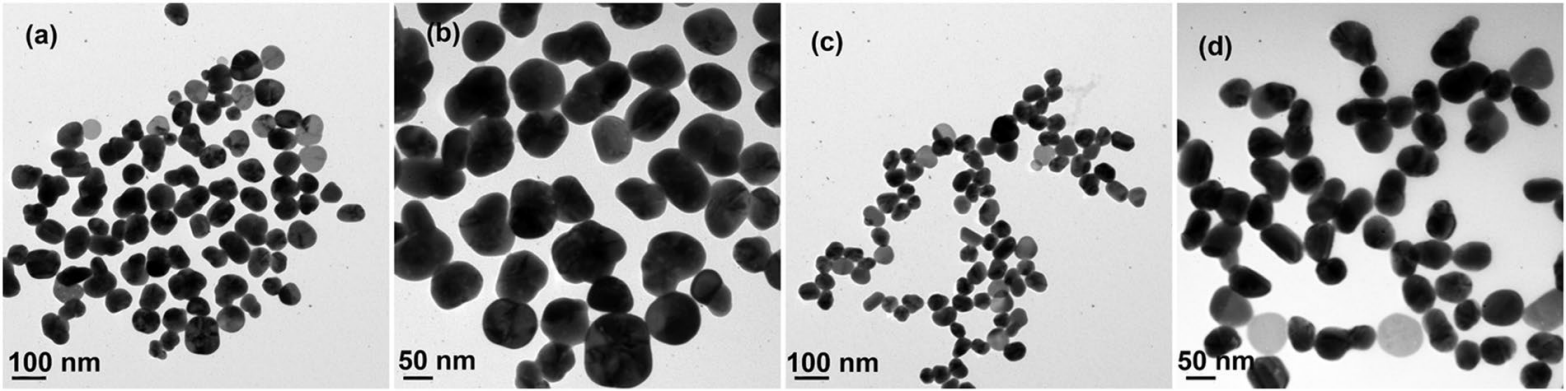
TEM images of Ag NMs synthesized by (**a**,**b**) adding nothing and (**c**,**d**) adding bare Ag NSs without polymer shell as seed in the precursor solution, respectively.

The effect of the added Ag NS@PSPAA seed on the mushroom-like nanostructure was then studied by doing the control experiments of adding nothing and adding bare Ag NSs without polymer shell as seed in the precursor solution, respectively. The synthesis procedures were similar to that described above. In the absence of seeds, irregular Ag NPs with the sizes of 50–100 nm appear as presented in Fig. 5a,b. Their sizes are same to those of the caps of Ag NMs (Fig. 2g,h). When only bare Ag NSs were used as seeds, however, the seeds grew bigger together with the formation of nanoplates due to the existence of both heterogeneous and homogeneous nucleation as illustrated in Fig. 5c,d. The second grown nanoplates are slightly larger than those NPs grow from the initial seeds. It can be hypothesized that without the forbidden of the polymer shell, the second added Ag can nucleate and grow freely on the surface of the bare Ag seeds and form uniform shells, which of course cannot be seen clearly due to the same metal type without obvious contrast difference. Avoiding the overgrowth on only one site, the final NPs will present slightly smaller diameter, which can also be attributed to the consumption of Ag during the simultaneous homogeneous nucleation process. Owing to the presence of sodium citrate, the Ag NPs come from the homogeneous nucleation show a flat shape. Another surprising phenomenon lies in that there is nearly no homogeneous nucleation resulted NPs in the preparation of NMs, which is probably because of the high affinity of Ag ions to the large amount of COO^−^ group on the surface of the polymer shell. Consequently, nearly all the Ag nucleate and grow on the surface of the Ag seeds through the polymer shell leading an extremely pure sample of NMs. As a result, a conclusion can be given that the final structure of the sample is critically dependent on the seed of polymer encapsulated Ag NPs.

**Figure 6:**
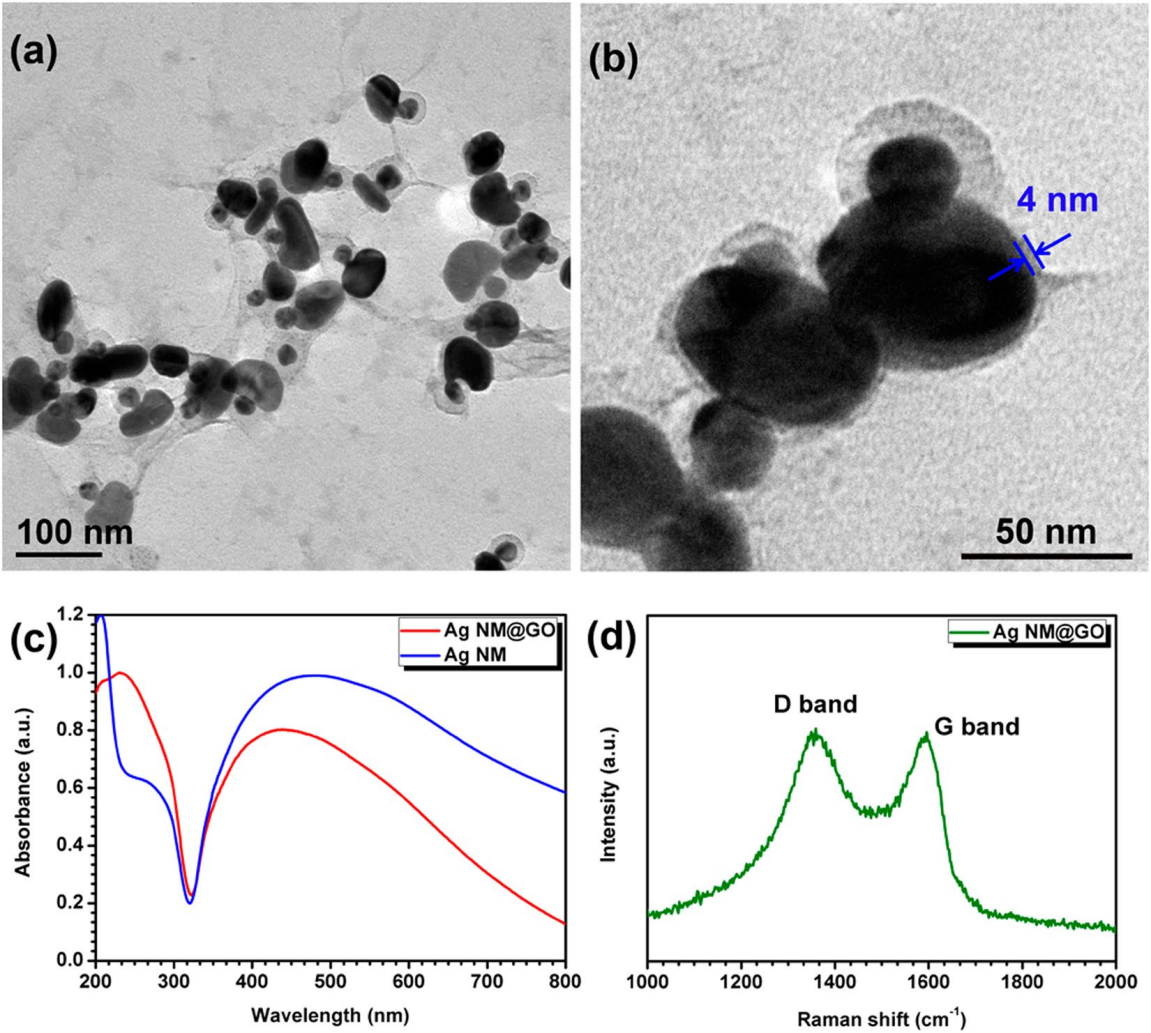
(**a**,**b**) TEM images of GO-wrapped Ag NMs, (**c**) absorption spectra of Ag NMs and GO-wrapped Ag NMs, and (**d**) Raman spectrum of GO-wrapped Ag NMs.

After successfully prepared the Ag NMs, we then modified them with positively charged groups to realize the wrapping of negatively charged GO sheets around them. Cysteamine was selected as the linking ligand because that the thiol groups from cysteamine can readily attach to the surface of the Ag NMs while the amino groups extend outwards to attract the negatively charged GO sheets and cause them to wrap around nearly all the NMs as shown in Fig. 6a. A TEM image of larger magnification in Fig. 6b further shows that GO shells with diameter of 5 ± 2 nm decorated on the surface of nearly all the Ag NMs, realizing a hybrid nanostructure. The absorption spectrum of the Ag NMs wrapped by GO was then compared with that of bare NMs. As shown in Fig. 6c, besides the broad LSPR band of Ag NMs at 475 nm, new obvious peak at 230 nm appears, which is attributed to the π-π^*^ electron transition of the C=O band^35^. Figure 6d further presents the Raman spectra of GO-wrapped Ag NMs without adding Raman ligand. The nanocomposite exhibits two characteristic peaks of GO at 1352 (D band) and 1591 (G band) cm^−1^, which could be attributed to the symmetry A_1g_ vibration mode and the E_2g_ vibration mode of sp^2^ carbon atoms, respectively^43^. All these results indicated that GO was successfully wrapped on the Ag NMs.

**Figure 7:**
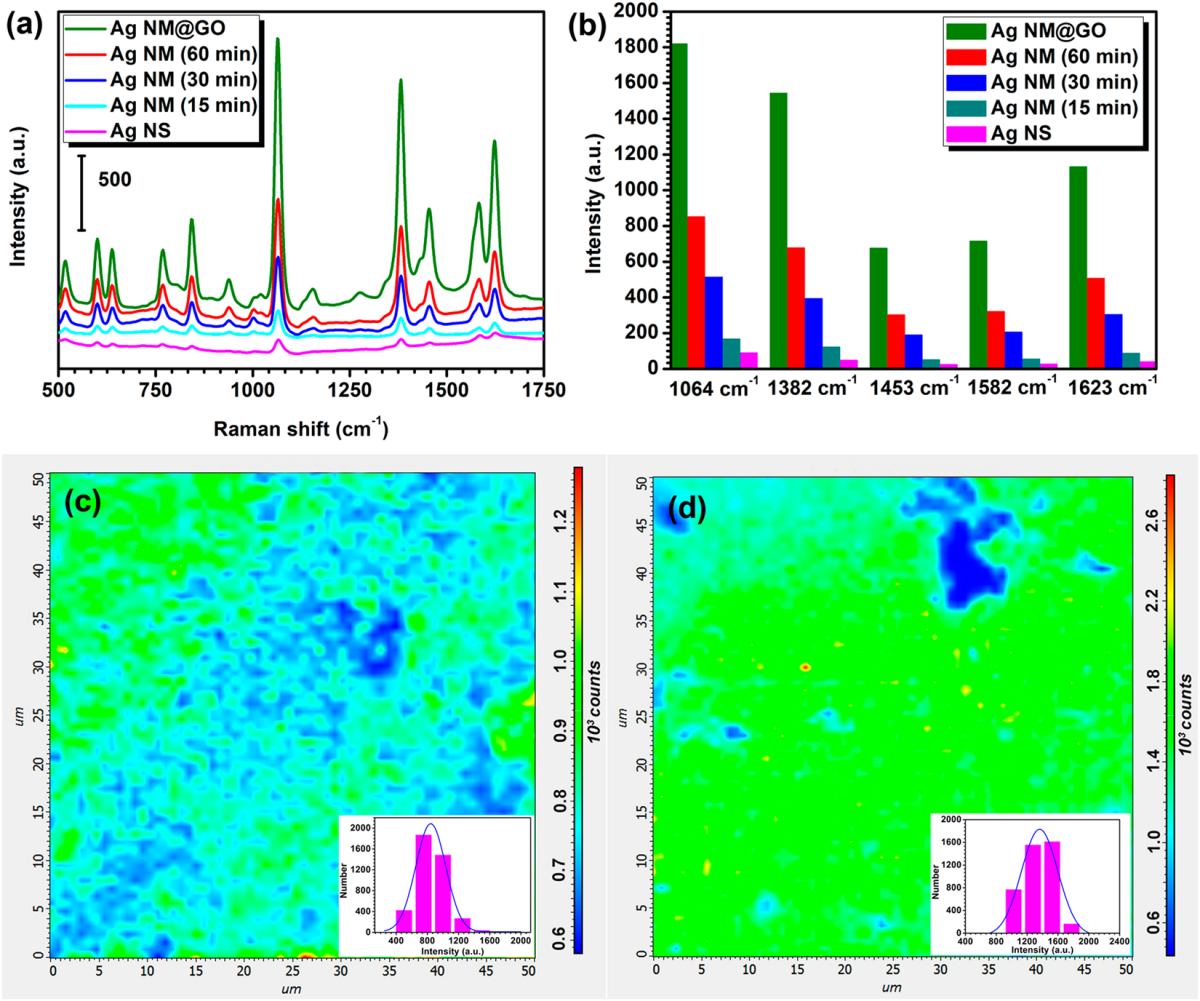
(**a**) SERS spectra of 2NT molecules in Ag NS@PSPAA, Ag NMs (15 min), Ag NMs (30 min), and Ag NMs (60 min), (**b**) the corresponding histogram of SERS peak intensities at five typical Raman shifts from these Ag NPs, and Raman maps (50 μm × 50 μm) on (**c**) Ag NMs and (**d**) GO-wrapped Ag NMs with the Raman band at 1382 cm^−1^ (the insets of (**c**) and (**d**) are the corresponding distributions of Raman intensity from Ag NMs and GO-wrapped Ag NMs).

As it has been reported, optical properties of plasmonic nanostructures are strongly dependent on their structural parameters and dielectric environment. Therefore, we then investigated the variation of the SERS spectra of the as-prepared nanostructures depending on the size of the mushroom cap and the existence of wrapped GO or not. Figure 7a displays the representative SERS spectra of 2NT molecules from these samples. Some characteristic bands of 2NT were detected with strong intensities, suggesting the exceptional SERS enhancement. The relative SERS intensities of the Ag NMs increased with the extension of the reaction time due to the growth of the outer Ag cap. Compared with the initial Ag NS@PSPAA, the finally obtained Ag NMs exhibit much higher SERS intensity with enhancement ratios of 2.67, 2.94, 2.02, 2.50, and 3.89 at 1064, 1382, 1453, 1582, and 1623 cm^−1^ as shown in Fig. 7b. It has been reported that ultra-small interparticle gap with the size of around 10 nm can generate a great local electromagnetic field terminated as hotspot due to electromagnetic coupling. In the present case, the Raman ligand was exactly encapsulated in the middle polymer shell between the inner and outer Ag layers, where is the major source of the hotspot. As a consequence, such an enhancement of SERS signal can be partially attributed to the formation of hotspots. Another possible reason for the increase of SERS intensity with the growth of Ag cap is resonant enhancement with the shifting of LSPR band to the excitation wavelength. The importance of an effective wrapping of GO around the Ag NMs for obtaining a much more enhanced SERS effect is also shown in Fig. 7a,b. The signals of 2NT completely overwhelm those of GO, so all the bands are related to molecular vibrations of Raman molecules. After encapsulated by GO, the SERS signal from Ag NMs (60 min) was further enhanced by around 2 fold, which means the SERS enhancement ratio of the GO-wrapped Ag NMs to the initial Ag NS@PSPAA was about 4–8.

Other than the high sensitivity, the homogeneity of the Raman signals from the SERS-active substrate is usually another major concern in the quantitative detection. In this work, a point-by-point Raman mapping was recorded on the random selected 50 × 50 µm^2^ area with a total of 4096 measurement points on the substrate of bare Ag NMs or those wrapped with GO, respectively. Figure 7c shows the Raman intensity mapping of the Raman band at 1382 cm^−1^ from the bare Ag NMs. Most of the areas of the substrate generate intense SERS signals with intensity from 800 to 900, represented by green color. After being further quantitatively analyzed, the corresponding distribution of SERS intensity shows the relative standard derivation (RSD) as 19.86% (the inset of Fig. 7c). The Raman intensity mapping of the same band at 1382 cm^−1^ from the GO wrapped ones was then obtained as shown in Fig. 7d. The SERS intensity increases to around 1800 and the corresponding RSD was calculated as 13.22% (the inset of Fig. 7d). The relative smaller dispersion of detection signals at around 10% confirms the advantages of the GO-wrapped Ag NMs as a more reliable and homogeneous SERS substrate. This comparison confirms that GO decoration has greatly enhanced the homogeneity of the SERS substrate due to its outstanding and unique chemical properties.

**Figure 8:**
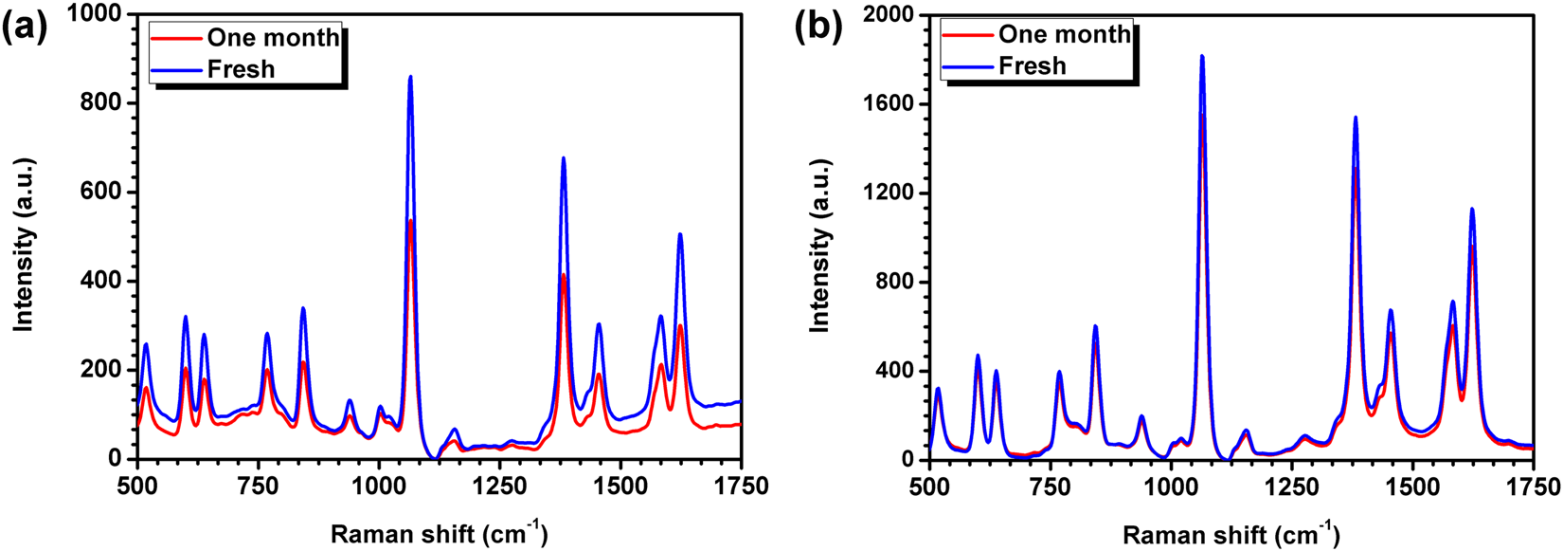
Measured SERS spectra from freshly synthesized (**a**) Ag NMs and (**b**) GO-wrapped Ag NMs and those were exposed to air for one month.

According to the literature, wrapped GO can also serve as a protective layer that prevents the Ag nanostructures from oxidation. The temporal stability of SERS signal from the GO-wrapped Ag NMs was therefore investigated. Figure 8a,b show the SERS spectra from the as-synthesized bare Ag NMs and GO-wrapped ones by exposing them to ambient air for 0 day and one month, respectively. For the bare Ag NMs, the characteristic peak intensity at the same spectral position shows a drastic drop, which decreases from 860 to 537 at 1064 cm^−1^, from 677 to 415 at 1382 cm^−1^, from 304 to 191 at 1455 cm^−1^, from 322 to 212 at 1582 cm^−1^, and from 506 to 301 at 1622 cm^−1^, after one month. The corresponding decrease ratio is 37.56%, 38.70%, 37.17%, 34.16%, and 40.51%, respectively. All the decrease ratios are larger than 30%, demonstrating the serious oxide instability of the bare Ag NMs when they were exposed to air. In contrast, for the GO-wrapped one, the SERS signal intensity decreases from 1818 to 1552 at 1064 cm^−1^, from 1542 to 1311 at 1382 cm^−1^, from 675 to 572 at 1455 cm^−1^, from 715 to 605 at 1582 cm^−1^, and from 1131 to 962 at 1622 cm^−1^, respectively. The corresponding decrease ratio was calculated as 14.63%, 14.98%, 15.26%, 15.38%, and 14.94%, respectively. Such decreases are probably due to the fact that the GO shell is not uniform as shown in Fig. 6a,b, some exposed parts of the Ag NMs may also be oxidized. However, the relatively low decrease ratios at around 15% indicate the excellent long-term stability of the GO-wrapped Ag NMs, which is significantly important for their practical application in SERS-based biological detection.

To enhance the SERS signals of Raman molecules, one of the effective strategies is embedding them between metal (or polymer) shell and metal core^44–46^. The Raman molecules have to be embedded between Ag core and polymer shell during the synthesis. Therefore, it is indeed a little difficult to detect other targeted molecules once such a type of core-shell nanostructure has been prepared. Although this method has limitation in detecting molecules, it could provide an alternative direction for engineering “hot” SERS nanostructures and for gaining new insights into SERS theory^46^. Particularly, such SERS-active nanostructure with a much clean surface (without attached Raman molecules) can also be easily modified with antigen to form an immune probe and used in SERS-based immunoassay to detect tumor markers^47^.

## Conclusions

In conclusion, Ag NMs were prepared by a seed-mediated growth method using core-shell Ag NS@PSPAA as core under the assistance of surfactant sodium citrate. A temporal dependent experiment was performed to investigate the growth process of the Ag NMs and NMs with caps of different size were obtained. The growth mechanism of this novel Ag nanostructure was further studied by changing the Ag seed from NS to nanocube and nanorod, followed by the reduction of AgNO_3_ with bare Ag NSs as seed or without seed. An enhancement ratio of SERS signal as 2.5 was observed from the final Ag NMs compared with the initial Ag NS@PSPAA due to the existence of hotspot in the polymer gaps and the resonant enhancement. The further encapsulation of a multilayer GO shell on the Ag NM leaded to a much better SERS property. The SERS signals from the GO-wrapped Ag NMs were 2 times higher than those from the bare NMs. The RSD value of the SERS intensity decrease from 19.86% to 13.22% with the coating of GO. After being stored for one month, GO-wrapped Ag NMs exhibited a more stable SERS signal with intensity decrease ratio of only 15%. This work represents the fabrication of GO-wrapped Ag NMs with intense, homogeneous, and stable SERS signals, which may open a new avenue for rationally designing plasmonic–graphene hybrid structures.

## Electronic supplementary material

Supplementary Info
