# Supplementary material for: Seed-mediated synthesis and SERS performance of graphene oxide-wrapped Ag nanomushroom
Source: Sci Rep. 2017 Aug 29;7:9795. doi: 10.1038/s41598-017-10262-9 (PMC5574994; doi:10.1038/s41598-017-10262-9)
Supplement: Supplementary file 1 — Supplementary Info [file 41598_2017_10262_MOESM1_ESM.doc]

**Supplementary materials to**

**Seed-mediated synthesis and SERS performance of graphene oxide-wrapped Ag nanomushroom**

Tao Jiang^1*^, Xiaolong Wang^1^, Shiwei Tang^1^, Jun Zhou^1^, Chenjie Gu^1^, and Jing Tang^2^

*^1^**Institute of Photonics, Department of Microelectronic Science and Engineering, Faculty of Science,* *Ningbo University, Ningbo 315211, P. R. China*

*^2^Institute of Physics,* *Ningbo University of Technology, Ningbo 315016, P.R. China*

**Synthesis of citrate-stabilized seed NPs**

In a representative synthesis, a 50 mL flask was charged with 1.47 mg of sodium citrate and 1.97 mg of HAuCl_4_·xH_2_O which were dissolved in 20 mL water. Then, 0.6 mL of ice-cold NaBH_4_ solution (0.1 M) was added with vigorous stirring. The solution turned from orange-yellow to brownish-red, indicating the formation of Au NPs. The average size of the resulting Au NPs was 3-4 nm according to the literature^1^.

**Encapsulation of Ag NSs with PSPAA**

In a typical synthesis, an Ag NS solution (1.0 mL) was concentrated to 10 μL by centrifugation at 16,000 g for 20 min. To the deep brownish-yellow suspension collected at the bottom of tube, 820 μL DMF was added, followed by 2NT in DMF (5 μL, 10 mg/mL) and incubated for 1 h. Then, PSPAA in DMF solution (80 μL, 8 mg/mL) and 200 μL H_2_O were added into the above solution. The total volume of the final mixture solution was 1.1 mL, where the DMF/H_2_O volume ratio was 4.5 and the PSPAA concentration was 28.5 μM. The mixture was heated to 110 °C for 2 h in the oil bath and then slowly cooled down to room temperature. To isolate the NPs, the product solution was diluted (300 μL diluted by 4200 μL water) to reduce the DMF content and de-swell the polymer shells. This transforms the PS layer into a rigid and glassy state and traps the NSs modified by ligand (2NT). The product solution was then centrifuged at 14,000 rpm for 30 min to remove the supernatant.

**References**

1. Jana, N. R., Gearheart, L. & Murphy, C. J. Seeding growth for size control of 5−40 nm diameter gold nanoparticles. *Langmuir* **17**, 6782–6786 (2001).

**Figure Legends**


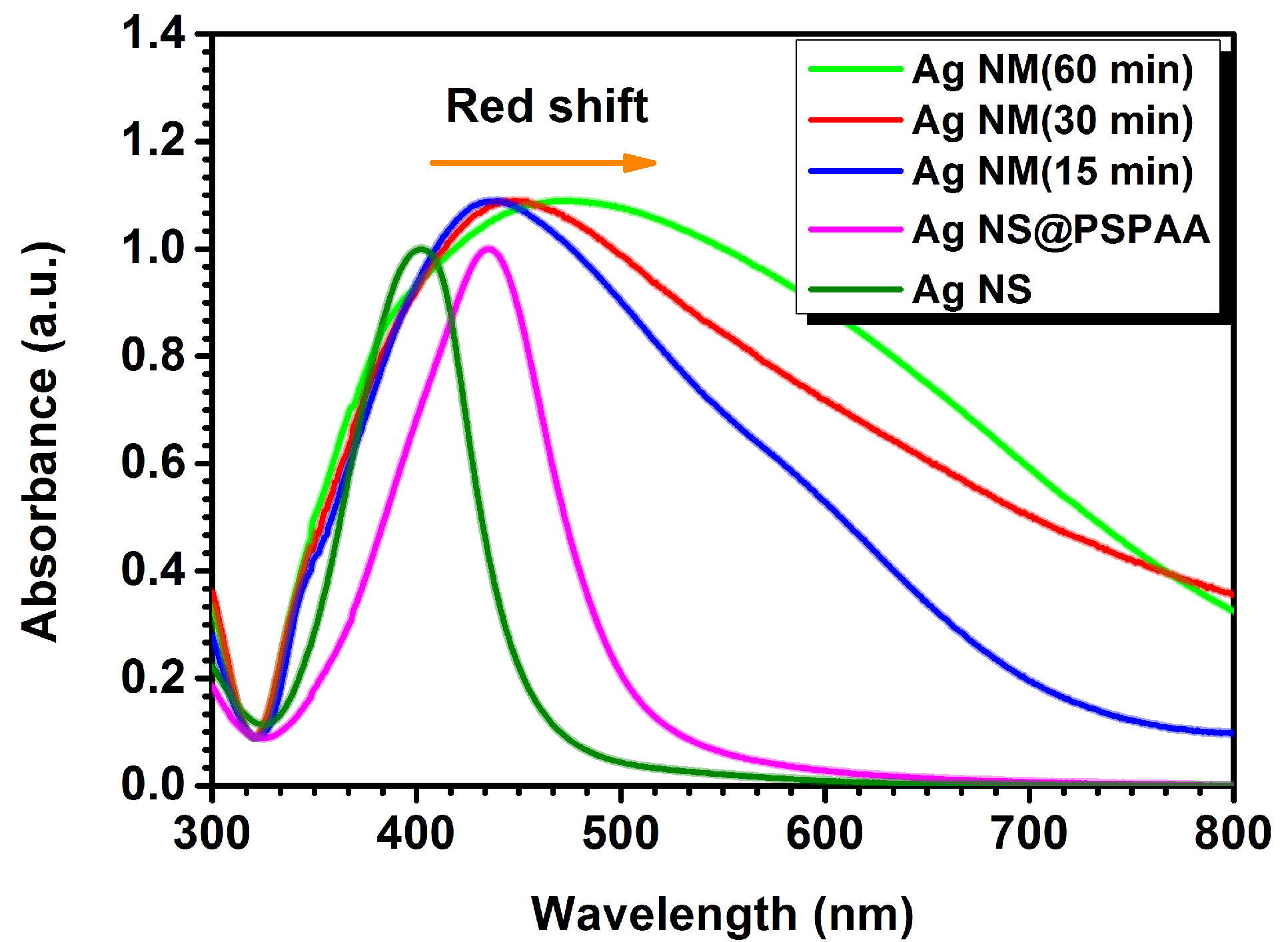


Figure. S1 Absorption spectra of Ag NSs, Ag NS@PSPAA, Ag NMs (15 min), Ag NMs (30 min), and Ag NMs (60 min).


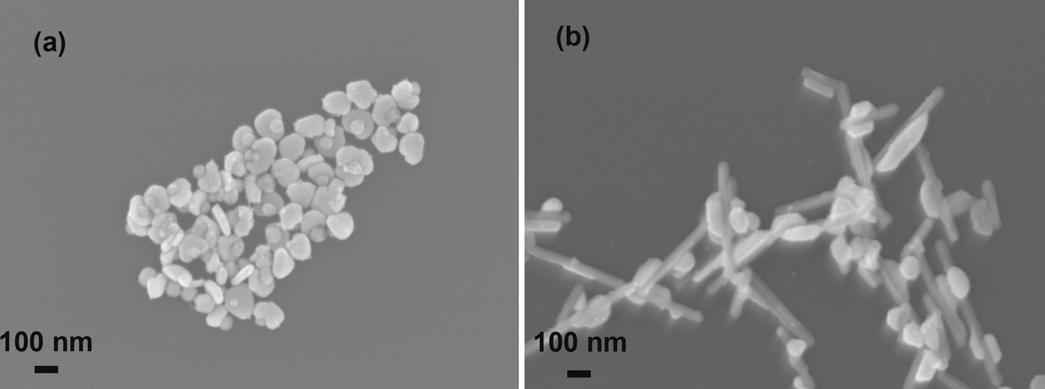


Figure. S2 SEM images of (a) Ag nanocube@PSPAA mediated Ag NMs and (b) Ag nanorod@PSPAA mediated Ag NMs.
